# Association of Optical Coherence Tomography With Longitudinal Neurodegeneration in Veterans With Chronic Mild Traumatic Brain Injury

**DOI:** 10.1001/jamanetworkopen.2020.30824

**Published:** 2020-12-22

**Authors:** Casey S. Gilmore, Kelvin O. Lim, Mona K. Garvin, Jui-Kai Wang, Johannes Ledolter, Alicia L. Fenske, Carolyn L. Gentz, Julie Nellis, Michael T. Armstrong, Randy H. Kardon

**Affiliations:** 1 Minneapolis VA Healthcare System, Minneapolis, Minnesota; 2 Defense and Veterans Brain Injury Center, Minneapolis, Minnesota; 3 Department of Psychiatry, University of Minnesota, Minneapolis; 4 Center for the Prevention and Treatment of Visual Loss, Iowa City VA Healthcare System, Iowa City, Iowa; 5 Department of Electrical and Computer Engineering, University of Iowa, Iowa City; 6 Department of Business Analytics and Department of Statistics and Actuarial Science, University of Iowa, Iowa City; 7 Department of Ophthalmology and Visual Sciences, University of Iowa, Iowa City

## Abstract

**Importance:**

Mild traumatic brain injury (TBI) may predispose individuals to progressive neurodegeneration.

**Objective:**

To identify evidence of neurodegeneration through longitudinal evaluation of changes in retinal layer thickness using optical coherence tomography in veterans with a history of mild TBI.

**Design, Setting, and Participants:**

This longitudinal cohort study evaluated veterans who were receiving services at the Minneapolis Veterans Affairs Health Care System. Symptomatic or mild TBI was diagnosed according to the Mayo TBI Severity Classification System. Participants in the age-matched control group had no history of TBI. Participants with any history or evidence of retinal or optic nerve disease that could affect retinal thickness were excluded. Data analysis was performed from July 2019 to February 2020.

**Exposures:**

The presence and severity of mild TBI were determined through consensus review of self-report responses during the Minnesota Blast Exposure Screening Tool semistructured interview.

**Main Outcomes and Measures:**

Change over time of retinal nerve fiber layer (RNFL) thickness.

**Results:**

A total of 139 veterans (117 men [84%]; mean [SD] age, 49.9 [11.1] years) were included in the study, 69 in the TBI group and 70 in the control group. Veterans with mild TBI showed significantly greater RNFL thinning compared with controls (mean [SE] RNFL slope, −1.47 [0.24] μm/y vs −0.31 [0.32] μm/y; *F*_1,122_ = 8.42; *P* = .004; Cohen *d* = 0.52). Functionally, veterans with mild TBI showed greater declines in visual field mean deviation (mean [SE] slope, −0.09 [0.14] dB/y vs 0.46 [0.23] dB/y; *F*_1,122_ = 4.08; *P* = .046; Cohen *d* = 0.36) and pattern standard deviation (mean [SE] slope, 0.09 [0.06] dB/y vs −0.10 [0.07] dB/y; *F*_1,122_ = 4.78; *P* = .03; Cohen *d* = 0.39) and high spatial frequency (12 cycles/degree) contrast sensitivity compared with controls. Cognitively, there was a significantly greater decrease in the number of errors over time during the Groton Maze Learning Test (GMLT) in controls compared with veterans with mild TBI (mean [SE] slope, −9.30 [1.48] errors/y vs −5.23 [1.24] errors/y; *F*_1,127_ = 4.43; *P* = .04; Cohen *d* = 0.37). RNFL tissue loss was significantly correlated with both worsening performance on the GMLT over time (Spearman ρ = −0.20; *P* = .03) and mild TBI severity (Spearman ρ = −0.25; *P* = .006). The more severe the mild TBI (larger Minnesota Blast Exposure Screening Tool severity score), the faster the reduction in RNFL thickness (ie, the more negative the slope) across time.

**Conclusions and Relevance:**

This cohort study found longitudinal evidence for significant, progressive neural degeneration over time in veterans with mild TBI, as indicated by greater RNFL tissue loss in patients with mild TBI vs controls, as well as measures of function. These results suggest that these longitudinal measures may be useful biomarkers of neurodegeneration. Changes in this biomarker may provide early detection of subsequent cognitive and functional deficits that may impact veterans’ independence and need for care.

## Key Points

**Question:**

Do veterans with a history of mild traumatic brain injury show greater neurodegeneration over time compared with control veterans with no history of head injury?

**Findings:**

In this longitudinal cohort study of 139 veterans with and without a history of mild traumatic brain injury, mild traumatic brain injury was associated with significantly greater thinning of the retinal nerve fiber layer over time.

**Meaning:**

These findings suggest that structural neural loss in the visual system, as evidenced by thinning of the retinal nerve fiber layer, may be a useful biomarker of neurodegeneration following chronic mild traumatic brain injury.

## Teaser

This cohort study examines neurodegeneration through longitudinal evaluation of changes in retinal layer thickness using optical coherence tomography in veterans with a history of mild traumatic brain injury (TBI).

## Introduction

Although traumatic brain injury (TBI) has long been considered a static event, it is better classified as a chronic disease process.^1^ Those with a history of TBI, including mild TBI, are at greater risk for neurodegenerative diseases, such as Alzheimer disease, Parkinson disease, and chronic traumatic encephalopathy, the last of which may occur after repetitive mild TBI or a single moderate-to-severe TBI.^1,2,3,4,5^ It is suspected that mild TBI may initiate a process of persistent neuroinflammation and long-term gray and white matter atrophy, leading to progressive neural degeneration over time. Several large studies have shown an association between a history of TBI and an increased risk of Alzheimer disease and Parkinson disease,^6,7,8^ even in individuals with no known cognitive impairments after TBI.^9^

These findings raise important questions about the long-term consequences of mild TBI, even in the absence of dysfunction following acute injury. Biomarkers may predate the development of cognitive and functional deficits by many years.^10^ Longitudinal studies can provide critical information on the temporal arc between the earliest biomarker changes and the subsequent cognitive and functional deficits impacting independence and quality of life.

Optical coherence tomography (OCT) is a noninvasive imaging technology used in ophthalmology to easily and rapidly examine and quantify the layers of the retina with high resolution (at the micrometer level), accuracy, and reproducibility.^11,12^ The association of OCT measures of retinal layer thinning with the degree of central nervous system (CNS) neurodegeneration has been well-established in disorders such as Alzheimer disease, Parkinson disease, and multiple sclerosis.^13,14,15,16,17,18,19,20^ OCT has been used to detect neurodegeneration in preclinical models of TBI^21,22,23^ and in a few human studies,^24,25,26,27,28^ but longitudinal studies have been lacking. The current study aimed to identify evidence of neurodegeneration through longitudinal evaluation of structural and functional changes in the visual nervous system and CNS in veterans with a history of mild TBI.

## Methods

### Participants

The study was approved by the Minneapolis VA Health Care System institutional review board. Written informed consent was obtained from all participants. This study follows the Strengthening the Reporting of Observational Studies in Epidemiology (STROBE) reporting guideline.

Participants were veterans receiving services in the Minneapolis VA Health Care System. The diagnosis of mild TBI was based on the Mayo TBI Severity Classification System.^29^ Any veteran with existing retinal or optic nerve conditions that could alter the thickness of the retinal nerve fiber layer (RNFL) over time were excluded. See the eAppendix, eFigure 1, eFigure 2, and eFigure 3 in the Supplement for more information on participant recruitment, inclusion criteria, and study timeline.

Participants completed a clinical interview at their baseline visit that included the Mini-International Neuropsychiatric Interview,^30^ Alcohol Use Disorders Identification Test,^31^ and the Minnesota Blast Exposure Screening Tool (see the eAppendix in the Supplement for a description of mild TBI determination and severity scoring, as well as characteristics of the mild TBI group).^32^ All outcome measures were assessed at 3- to 6-month intervals.

### Optical Coherence Tomography

OCT imaging was performed in each eye without pupil dilation using the Spectralis OCT1 (Eye Explorer software version 1.9; Heidelberg Engineering). The mean peripapillary RNFL thickness was determined by the instrument segmentation software of the circular scan. The mean thickness of the ganglion cell layer complex (GCL-IPL) in the central 8° was segmented from an elliptical volume macular scan using the Iowa Reference Algorithms (Retinal Image Analysis Lab; Iowa Institute for Biomedical Imaging) (eAppendix in the Supplement). RNFL and GCL-IPL complex thickness values were assessed over time for the right eye, left eye, and averaged across right and left eyes for each time point, per participant (eAppendix in the Supplement).

### Visual Function Measures

#### Visual Acuity

Best-corrected, high-contrast (100% contrast), distant equivalent visual acuity was measured in each eye using the Functional Vision Analyzer system (Stereo Optical Co Inc). The outcome acuity score was the number of letters read correctly on an internally projected chart (eg, a change in 5 letters = 0.1 logMAR change in acuity).

#### Contrast Sensitivity

Contrast was a threshold measure for detecting the orientation of sinusoidal gratings at the lowest contrast at 5 spatial frequencies: 1.5, 3, 6, 12, and 18 cycles per degree, using the aforementioned Functional Vision Analyzer and as detailed in the eAppendix in the Supplement. For each spatial frequency, 9 sine-wave gratings in 0.15 log contrast sensitivity decrements were presented (eg, a negative slope of 2 units/y in contrast sensitivity score would mean that there was a loss of 0.3 log contrast sensitivity).

#### Visual Field

Automated visual field sensitivity in each eye was tested with the Humphrey Frequency Doubling Perimeter (Zeiss Meditec), using a 10-2 threshold program to assess the central 10° (eAppendix in the Supplement).^33^ The 2 outcome measures were the decibel mean deviation (a center-weighted mean of all of the locations tested) from the age-matched normative database (negative values are worse than age-matched normal eyes), and pattern standard deviation, a decibel measure of regional asymmetry (higher values represent more focal loss, and lower values represent either no loss or diffuse loss).

### Cognitive Measures

Cognitive ability was measured using 5 standard tests from the CogState battery (CogState Lt)^34^: Detection, a test of psychomotor processing speed; Identification, an attentional test; One-Back Task, which assesses working memory; One Card Learning, which assesses short-term visual learning and memory; and Groton Maze Learning Test (GMLT), which measures executive function using a maze learning paradigm. See the eAppendix in the Supplement for more details.

### Statistical Analysis

#### Longitudinal Changes of Outcome Measures

A linear regression model, using the lm() function in R statistical software version 3.6 (R Project for Statistical Computing), was used to estimate the intercept and slope for outcome measures over time on those participants who had 3 or more time points of data. The Cook distance method of identifying outliers was used (eAppendix in the Supplement).^35^ To compare changes in outcome measures between the TBI and control groups, we used the slopes calculated from the linear model as the dependent variable in a univariable analysis of variance after passing normality tests. An alternative statistical mixed-effects model of change over time in outcome variables was also used to analyze all observations over time in a single model.^36^ The model incorporates random variability for outcome-specific intercepts and slopes around the fixed-effects intercept and mean slopes of the respective treatment groups (control and mild TBI). An advantage of this model is that it accounts for the different number of measurements on each eye, whereas the previous analysis assumes equal precision on all slope estimates. The model can be fit with the PROC MIXED function of SAS statistical software version 9.4 (SAS Institute). Two-sided *F* tests with *P* < .05 were used to test for statistical significance.

#### Correlation Between Changes in OCT Retinal Thickness and Functional Outcome Measures

The correlation between slope of OCT retinal layer thickness and the following outcome measures was assessed using Spearman ρ correlation coefficient: mild TBI severity, time since most recent head injury, visual function slopes, and CogState slopes. Data analysis was performed from July 2019 to February 2020.

## Results

 A total of 139 veterans (117 men [84%]; mean [SD] age, 49.9 [11.1] years) were included in the study (69 in the mild TBI group and 70 in the control group). The groups were similar with regard to age, sex, education level, and prevalence of hypertension, diabetes, hazardous drinking or alcohol use disorder, and posttraumatic stress disorder (Table 1). The median (range) time since injury was 17 (0.5-59) years.

**Table 1.** Characteristics of the Sample

Characteristic	Participants, No. (%)		Test statistic (χ^2^ or *F*)	*P* value
	TBI (n = 69)	Control (n = 70)		
Age, mean (SD), y	50.0 (10.3)	49.9 (11.9)	0.001^a^	.97
Women	7 (10.1)	15 (21.4)	3.32^b^	.07
Education level, mean (SD)^c^	6.7 (1.8)	6.8 (1.8)	0.12^a^	.73
Hypertension	25 (36)	27 (39)	0.08^b^	.78
Diabetes	13 (19)	12 (17)	0.07^b^	.80
Hazardous drinking or alcohol use disorder	26 (38)	22 (31)	0.61^b^	.44
Posttraumatic stress disorder	6 (9)	4 (6)	0.46^b^	.50
TBI severity score, mean (SD)	2.3 (2.7)	NA	NA	NA

Abbreviations: NA, not applicable; TBI, traumatic brain injury.

^a^ *F* test was used for numeric variables.

^b^ χ^2^ test was used for categorical variables.

^c^ Education level was coded as follows: 1 = less than 8 years, 2 = some high school, 3 = graduated high school, 4 = general education diploma, 5 = work toward associate’s degree, 6 = completed associate’s degree, 7 = work toward bachelor’s degree, 8 = completed bachelor’s degree or higher, 9 = other.

### OCT Measures of Retinal Layer Thickness

There were a total of 124 participants (63 in the TBI group and 61 in the control group) who met the criteria for inclusion in the linear regression model calculation (ie, they had ≥3 time points of RNFL data). Reasons for having fewer than 3 time points of data included missed visits, technical difficulties with OCT equipment during a visit, and inability to collect usable data because of eye or other participant issues during a visit.

**Figure 1.**
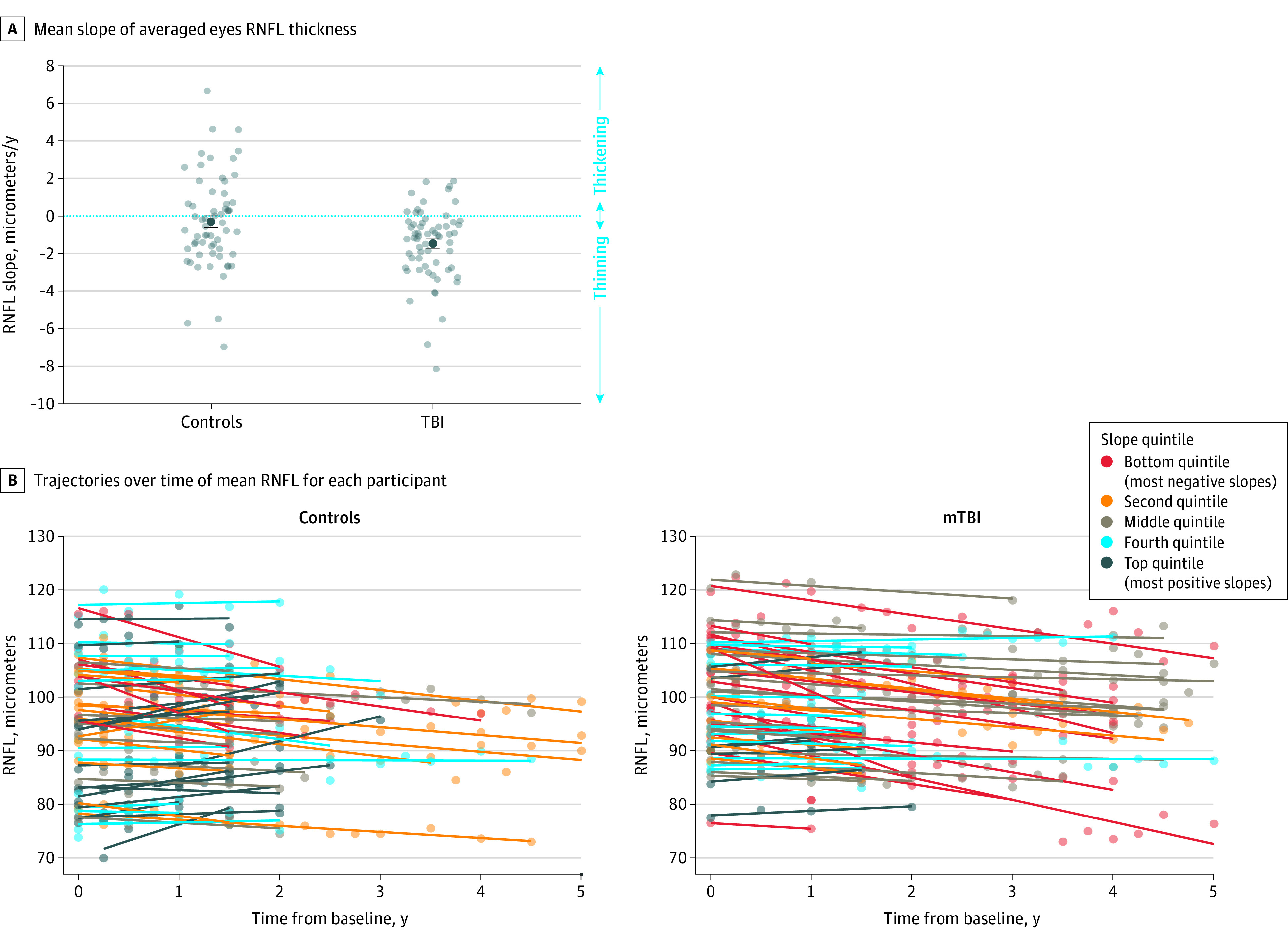
Mean Slope of Averaged Eyes Retinal Nerve Fiber Layer (RNFL) Thickness and Trajectories of Mean RNFL Thickness Over Time for Individual Participants A, Graph shows mean slope of averaged eyes RNFL (micrometers per year; dark dots with SE bars). Light dots show data for individuals in the mild traumatic brain injury (mTBI) and control groups. Groups significantly differed (mean [SE] RNFL slope, −1.47 [0.24] μm/y vs −0.31 [0.32] μm/y; *F*_1,122_ = 8.42; *P* = .004, Cohen *d* = 0.52). B, Graphs show trajectories of RNFL thickness over time for individual participants in mTBI and control groups; dots are time points and lines are linear fit lines for each participant. Colors represent the RNFL slopes grouped by quintiles: bottom quintile contains the most negative slopes, top quintile contains the most positive slopes.

The main outcome measure, RNFL change over time, showed significantly more thinning (loss of axons) per year in the mild TBI group compared with controls (mean [SE] RNFL slope, −1.47 [0.24] μm/y vs −0.31 [0.32] μm/y; *F*_1,122_ = 8.42; *P* = .004; Cohen *d* effect size = 0.52) (Figure 1). The mixed-effects model showed an even more significant result (difference in slope β, −1.80; *P* = .001) (Table 2). Interestingly, the baseline RNFL thickness at enrollment showed a significantly thicker mean (SE) RNFL for participants with mild TBI vs controls (98.4 [1.2] μm vs 93.8 [1.4] μm; *F*_1,135_ = 6.25; *P* = .01; Cohen *d* = 0.43), which was also confirmed by the intercept estimate using the mixed-effects linear model. There was no correlation between the time since TBI and the baseline RNFL thickness. Unlike RNFL, the change in thickness of the ganglion cell layer complex corresponding to the central 8° of vision was no different between controls and mild TBI groups.

**Table 2.** Visual Structure and Function Slope Measures in TBI and Control Subjects: Analysis of Variance and Mixed-Effects Model Results

Variable	Slope, mean (SE)		*F*	*P* value	Cohen *d*	Mixed-effects model	
	TBI	Controls				Difference in slope, β^a^^,^^b^	*P* value
OCT retinal nerve fiber thickness, μm/y (n = 63 TBI; n = 61 controls)							
Averaged eyes	−1.47 (0.24)	−0.31 (0.32)	8.42	.004^c^	0.52	−1.80	.001^c^
OD	−1.55 (0.27)	−0.01 (0.36)	11.77	<.001^c^	0.62	−1.50	.001^c^
OS	−1.16 (0.33)	−0.29 (0.52)	2.01	.16	0.26	−0.81	.02^c^
OCT ganglion cell layer complex, μm/y (n = 63 TBI; n = 66 controls)							
Averaged eyes	−0.17 (0.07)	−0.02 (0.09)	2.00	.16	0.25	0.08	.51
OD	−0.20 (0.08)	−0.11 (0.10)	0.46	.49	0.12	−0.08	.58
OS	−0.19 (0.06)	0.01 (0.10)	2.76	.10	0.29	−0.19	.16
Visual acuity in change, No. of letters correct per y (n = 60 TBI; n = 62 controls)^a^							
Averaged eyes	−0.71 (0.41)	0.36 (0.45)	3.08	.08	0.32	Not analyzed	
OD	−0.98 (0.50)	−0.10 (0.60)	1.25	.27	0.20	−1.50	.26
OS	−0.42 (0.48)	0.80 (0.53)	2.93	.09	0.31	−1.16	.05
Visual field mean deviation, dB/y (n = 60 TBI; n = 64 controls)							
Averaged eyes	−0.09 (0.14)	0.46 (0.23)	4.08	.046^c^	0.36	Not analyzed	
OD	−0.004 (0.18)	0.26 (0.26)	0.71	.40	0.15	−0.19	.46
OS	−0.14 (0.14)	0.66 (0.23)	8.43	.004^c^	0.52	−0.52	.03^c^
Visual field pattern standard deviation, dB/y (n = 60 TBI; n = 64 controls)							
Averaged eyes	0.09 (0.06)	−0.10 (0.07)	4.78	.03^c^	0.39	Not analyzed	
OD	0.06 (0.07)	−0.14 (0.08)	3.23	.07	0.32	0.12	.15
OS	0.11 (0.07)	−0.07 (0.07)	3.33	.07	0.33	0.11	.11
Contrast sensitivity (2 units/y = 0.3 log unit change) (n = 63 TBI; n = 62 controls)^d^							
1.5 cpd							
Averaged eyes	−1.91 (0.99)	0.20 (0.91)	2.48	.12	0.28	Not analyzed	
OD	−2.04 (1.03)	2.01 (1.03)	7.73	.006^c^	0.50	−3.14	.008^c^
OS	−1.48 (1.22)	−2.39 (1.37)	0.25	.62	0.09	0.56	.70
3 cpd							
Averaged eyes	0.44 (1.22)	0.31 (1.77)	0.003	.95	0.01	Not analyzed	
OD	1.61 (1.75)	1.69 (1.82)	0.001	.97	0.01	−1.55	.42
OS	−0.51 (1.26)	−0.81 (2.54)	0.01	.92	0.02	−0.53	.78
6 cpd							
Averaged eyes	−0.73 (1.82)	2.15 (2.06)	1.11	.29	0.19	Not analyzed	
OD	−1.08 (2.17)	2.74 (2.55)	1.31	.25	0.20	−3.86	.13
OS	0.23 (2.20)	1.73 (2.40)	0.21	.65	0.08	−2.19	.37
12 cpd							
Averaged eyes	−1.51 (0.93)	1.49 (0.84)	5.70	.02^c^	0.43	Not analyzed	
OD	−1.51 (1.10)	1.33 (1.04)	3.50	.06	0.33	−1.15	.39
OS	−1.55 (1.01)	1.78 (1.06)	5.15	.02^c^	0.41	−1.26	.30
18 cpd							
Averaged eyes	−0.30 (0.52)	0.41 (0.44)	1.08	.30	0.19	Not analyzed	
OD	−0.62 (0.81)	0.23 (0.54)	0.76	.39	0.16	0.008	.99
OS	0.02 (0.56)	0.37 (0.54)	0.21	.65	0.08	0.23	.69

Abbreviations: cpd, cycles per degree; OCT, optical coherence tomography; OD, oculus dexter (right eye); OS, oculus sinister (left eye); TBI, traumatic brain injury.

^a^ Visual acuity is measured in number of letters correct; a change in 5 letters = 0.1 logMAR acuity and 1 line of letters on an EDTRS chart. For example, a slope of 1 letter lost per year means that in 5 years, it would be expected that 1 line of letters would be lost (eg, 20/25 worsening to 20/30 visual acuity).

^b^ The test of β = 0 assesses whether the mean slopes are the same in the mixed-effects model. Change is defined as the difference from control. If change is negative, TBI slope is less than control slope.

^c^ Denotes statistical significance.

^d^ For each spatial frequency, 9 sine-wave gratings in 0.15 log contrast sensitivity decrements were presented (eg, a negative slope of 2/y would mean that there was a loss of 0.3 log contrast sensitivity).

### Visual Function Measures

Table 2 shows the analysis of variance results for change over time (slope) of the visual function measures: visual acuity, visual field mean deviation and pattern standard deviation, and contrast sensitivity at 5 spatial frequencies. Change over time of visual acuity (letter score per year) did not significantly differ between groups. Both visual field mean deviation and pattern standard deviation significantly differed between groups when both eyes were averaged together. The TBI group had a significantly more negative slope for mean deviation (mean [SE], −0.09 [0.14] dB/y vs 0.46 [0.23] dB/y; *F*_1,122_ = 4.08; *P* = .046; Cohen *d* = 0.36) and a significantly more positive slope for pattern standard deviation (mean [SE], 0.09 [0.06] dB/y vs −0.10 [0.07] dB/y; *F*_1,122_ = 4.78; *P* = .03; Cohen *d* = 0.39) compared with the control group, both of which indicate worsening visual field thresholds over time in the TBI group (eFigure 4 and eFigure 5 in the Supplement). To account for multiple comparisons in the contrast sensitivity data, we chose to control the false discovery rate (eAppendix in the Supplement). Contrast sensitivity testing showed significant changes over time in the right eye at the lowest spatial frequency of 1.5 cycles/degree (*F*_1,123_ = 7.73; *P* = .006; Cohen *d* = 0.50) and in the left eye (*F*_1,123_ = 5.15; *P* = .02; Cohen *d* = 0.41) and both eyes combined (*F*_1,123_ = 5.70; *P* = .02; Cohen *d* = 0.43) at the 12 cycles per degree spatial frequency (eFigure 6 in the Supplement).

### CogState Measures

There were a total of 130 participants (64 in the TBI group and 66 in the control group) who met the criteria for inclusion in the linear regression model calculation (ie, they had ≥3 time points of data) for the CogState tasks. Because 5 tasks were administered, we controlled for the false discovery rate using the same method as for the contrast sensitivity measures. Table 3 shows that, of all of the CogState measures, only reduction in total errors committed during the GMLT over time was significantly worse in the mild TBI group compared with the control group (mean [SE] slope, −5.23 [1.24] errors/y vs −9.30 [1.48] errors/y; *F*_1,127_ = 4.43; *P* = .04; Cohen *d* = 0.37) (eFigure 7 in the Supplement).

**Table 3.** CogState Cognitive Task Slope Measures Analysis of Variance Results

Task	Slope, mean (SE)		*F*	*P* value	Cohen *d*
	TBI (n = 64)	Controls (n = 66)			
Detection	0.01 (0.03)	0.03 (0.03)	0.26	.61	0.09
Identification	−0.0003 (0.03)	0.02 (0.01)	0.44	.51	0.12
One Card Learning	0.00003 (0.006)	0.0007 (0.007)	0.01	.94	0.01
One Back Task	0.00006 (0.004)	0.001 (0.007)	0.03	.87	0.03
Groton Maze Learning Test	−5.23 (1.24)	−9.30 (1.48)	4.43	.04^a^	0.37

Abbreviation: TBI, traumatic brain injury.

^a^ Denotes statistical significance.

### Correlation Between Changes in OCT Retinal Thickness and Functional Outcome Measures

Spearman ρ correlations showed that although it was not significantly correlated with time since injury in the TBI group (mean [SE] time since injury, 20.1 [1.8] years; Spearman ρ = 0.13; *P* = .32), RNFL slope was significantly correlated with mild TBI severity across the whole sample (Spearman ρ = −0.25; *P* = .006) (eFigure 8 in the Supplement). The more severe the mild TBI (larger Minnesota Blast Exposure Screening Tool derived severity score), the faster the reduction in RNFL thickness (ie, the more negative the slope) across time.

**Figure 2.**
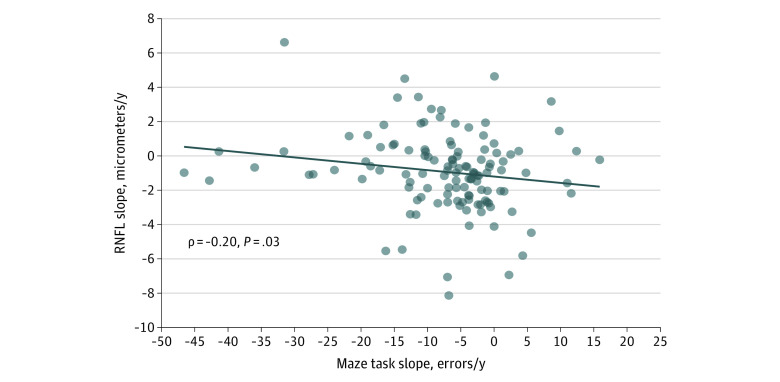
Scatter Plot Showing Significant Correlation Between Averaged Eyes Retinal Nerve Fiber Layer (RNFL) Thickness Change Over Time and Change Over Time in Total Errors During the Groton Maze Learning Test

RNFL thickness change over time (slope) was also significantly correlated with slope of total errors during the GMLT (Spearman ρ = −0.20; *P* = .03) (Figure 2). A greater reduction in errors made over time in the GMLT (greater negative slope) was associated with less reduction in RNFL thickness over time (smaller negative slope, or positive slope). No variables were significantly correlated with GCL-IPL change over time.

## Discussion

This study found a significant reduction in retinal axons over time as a biomarker of progressive neurodegeneration in veterans with chronic mild TBI. We also found evidence for progressive loss of visual function (contrast and visual field sensitivity) and CNS function (lack of improvement of error score on the GMLT) in participants with mild TBI. Furthermore, faster RNFL thinning correlated with greater severity of mild TBI and less improvement in cognitive performance over time in the GMLT.

Thinning of RNFL as a sign of neurodegeneration is not a new concept. Examples have been reported in patients with Alzheimer disease, pre–Alzheimer disease with cognitive impairment,^37^ Parkinson disease,^38,39^ cognitive impairment,^39^ and multiple sclerosis, including patients who have never had an episode of optic neuritis.^40^ OCT studies in rodent models of TBI have shown thinning of the RNFL in a blast model,^21^ blast combined with a genetic model of Alzheimer disease,^22^ and in a concussive model.^23^ Reports of a thinner RNFL after head trauma have also been published in 3 human studies.^24,25,26^ These results demonstrate that change in retinal layer thickness as measured with OCT is a sensitive and precise biomarker of loss of neurons, but, as of yet, is nonspecific for differentiating neurological disorders. It is, however, an easily administered and sensitive tool to facilitate detection and monitoring of neurodegeneration.

In this study, we found evidence for both decreases and increases in RNFL over time in the control and mild TBI groups. Although the mild TBI group showed a greater negative slope (thinning over time), there were individual participants in both groups with positive slopes over time. This was unexpected because axonal layer thickness is thought to be stable or, in the case of progressive neural degeneration, decreases over time. Although measurement variability of thickness can occur as a result of factors influencing segmentation of the retinal layers, such as signal strength or neighboring blood vessels, such factors would not explain a systematic trend of increases in thickness over time. The RNFL is not static; it contains axons and glial cells. Axon volume may be influenced by the characteristics of axoplasmic flow and status of the axon,^36,41,42,43,44,45^ which, in turn, may cause dynamic changes in the thickness of this layer. Changes in glial cells, including Mueller cells in the inner retina, could also explain an increase in RFNL over time due to gliosis. Future investigation is warranted to better understand the reason why some eyes show an increase in axon layer thickness over time.

We also found that the mild TBI group had a thicker RNFL at the time of enrollment compared with age-matched control veterans. Differences in axoplasmic flow, gliosis, and axial eye length are plausible explanations. We also found no significant correlation between time elapsed from TBI and baseline RNFL thickness or slope. Although there was no correlation, it is notable that our sample had a wide range of times since injury (median [range], 17 [0.5-59] years) and that some of the study patients were showing signs of chronic neurodegeneration years after their TBI event. This would argue for a nonlinear process of neural degeneration that does not necessarily start at the time of TBI and could be a result of neuroinflammation, the time course of which has not yet been clearly elucidated. Future analyses and studies will investigate this time course.

Although RNFL showed significantly greater thinning over time in mild TBI with a medium effect size, there was not a corresponding difference in thinning of the GCL-IPL over time. Ganglion cells are concentrated in the macula; their density and multilayer organization in the central 8° in the macula provide sufficient dynamic range (40-100 μm) to detect thinning by OCT. Lack of progressive thinning of the GCL-IPL in the macula would suggest relative sparing of progressive neurodegeneration in the central 8° visual field, which may explain why these patients did not appreciate subjective changes in visual function over time. Currently, OCT is not precise enough to segment and detect significant changes in the GCL-IPL outside of the macula where the soma are only 1 layer thick, offering little dynamic range of thickness. The reduction in the mean peripapillary RNFL thickness over 360° implies diffuse damage, corresponding mainly to the peripheral visual field.^46^ Further regional analysis of the RNFL is planned, which may elucidate whether certain axon bundles are more susceptible to neurodegeneration, similar to glaucoma.

The greater RNFL thinning we found in the mild TBI group may be due to retrograde degeneration, including trans-synaptic retrograde degeneration^47,48^ resulting from damage to the postgeniculate visual radiations or visual cortex^28^; however, we cannot ascertain whether the initial damage was at the pregeniculate or postgeniculate location (or both) at this time. We are planning to quantify the spatial pattern of white and gray matter changes over time from magnetic resonance imaging studies on these cohorts to determine whether the RNFL thinning precedes or follows thinning of corresponding visual radiations and visual cortex in order to provide evidence for trans-synaptic anterograde vs retrograde degeneration in the CNS underlying post-TBI neurodegeneration.

The results of visual and cognitive functional tests also showed evidence for progressive loss, but to a lesser degree than structural loss, and only in some of the behavioral functions tested. Some behavioral measures of visual and cognitive function may be more sensitive to neurodegeneration than others (eg, low contrast acuity vs high contrast acuity^49^) or may lag behind structural measures, and trends may not be significantly different from normal variations over the duration of this study. In glaucoma, which is the most common neurodegeneration of the retina and optic nerve, it is common to find evidence for structural loss of inner retinal thickness before corresponding functional deficits can be detected.^50^ The same holds true for the cognitive tests. Simpler tests of reaction time and attention did not differ between groups over time, but a significant difference was found in the GMLT, a cognitive test of spatial working memory and error monitoring that is sensitive to longitudinal changes in cognitive ability.^51^ Improvement of the task over time was associated with less thinning in RNFL over time, suggesting that retinal axons could be used as a surrogate for loss of neurons in the CNS affecting executive function, warranting further study.

### Limitations

Some limitations must be considered when interpreting these results. First, the diagnosis of mild TBI relied on self-report of the incident and symptoms surrounding the head injuries. Findings have shown evidence for variable success in recall of head injury events, with better recall if there was less time between the event and time of recall, greater injury severity, and greater significance of the event to the individual or their family.^52,53^ Ideally, medical records could be used to corroborate head injury events; however, records review is not always possible or satisfactory, and many TBI events, particularly mild ones, were not treated medically. We opted for a comprehensive interview to assess TBI history, which has been shown to elicit superior recall compared with questionnaires.^54^ Second, the current study focused on mild TBI, and, although mild TBI is the most prevalent form of TBI, longitudinal studies of patients with moderate-to-severe TBI may better reveal the association of TBI with neurodegeneration over time. Furthermore, although we did not adjust for multiple tests across the various measurement domains, the results are consistent and demonstrate different manifestations of the same pathological process.

## Conclusions

The findings of this cohort study provide evidence for progressive neurodegeneration and functional loss over time in veterans with mild TBI. We found greater thinning of the RNFL in the retina in the mild TBI group compared with control veterans. We also found evidence of greater decline in functional measures of vision (visual field and contrast sensitivity) and cognition (GMLT) in mild TBI, with cognitive decline being associated with the RNFL thinning. These findings fit with theoretical models of the chronic effects of neurotrauma,^10^ with structural biomarkers showing abnormality first followed by functional abnormalities. Taken together, these findings suggest that the precision of OCT can be applied to identify individuals showing thinning of the axon layer of the inner retina as a marker for progressive neurodegeneration after mild TBI. Early identification of patients with neurodegeneration may provide a strategy for personalized medicine in which treatments that mitigate the consequences of neurodegeneration, including drugs and neuromodulation, could be instituted at a time that may preserve function.

## Notes

Supplement. **eAppendix.** Supplemental Methods **eFigure 1.** Frequency Distribution of the Time, in Years, Participants Were Enrolled in the Study **eFigure 2.** Frequency Distribution of the Number of Time Points of Data Collected for Each Participant **eFigure 3. **Frequency Distribution of the Number of Self-reported Blast (3a) and Nonblast (3b) Subconcussive Head Injury Exposures in the TBI Group **eFigure 4.** Mean Slope of Averaged Eyes Visual Field Mean Deviation (dB/year; Colored Dots With SE Bars) and Individual Subjects’ Slopes (Gray Dots) for Each Group **eFigure 5.** Mean Slope of Averaged Eyes Visual Field Pattern Standard Deviation (dB/Year; Colored Dots With SE Bars) and Individual Subjects’ Slopes (Gray Dots) for Each Group **eFigure 6.** Mean Slope of Averaged Eyes Contrast Sensitivity Score at 12 cpd (Score/Year; Colored Dots With SE bars) and Individual Subjects’ Slopes (Gray Dots) for Each Group **eFigure 7.** Mean Slope of Groton Maze Learning Test (GMLT) Total Errors (Errors/Year; Colored Dots With SE bars) and Individual Subjects’ Slopes (Gray Dots) for Each Group **eFigure 8.** Scatter Plot Showing Significant Correlation Between RNFL Thickness Change Over Time (Microns/Year) and mTBI Severity Score for the Whole Sample **eReferences**
