# Supplementary material for: Association of Optical Coherence Tomography With Longitudinal Neurodegeneration in Veterans With Chronic Mild Traumatic Brain Injury
Source: JAMA Netw Open. 2020 Dec 22;3(12):e2030824. doi: 10.1001/jamanetworkopen.2020.30824 (PMC7756235; doi:10.1001/jamanetworkopen.2020.30824)
Supplement: Supplement. — eAppendix. Supplemental Methods eFigure 1. Frequency Distribution of the Time, in Years, Participants Were Enrolled in the Study eFigure 2. Frequency Distribution of the Number of Time Points of Data Collected for Each Participant eFigure 3. Frequency Distribution of the Number of Self-reported Blast (3a) and Nonblast (3b) Subconcussive Head Injury Exposures in the TBI Group eFigure 4. Mean Slope of Averaged Eyes Visual Field Mean Deviation (dB/year; Colored Dots With SE Bars) and Individual Subjects’ Slopes (Gray Dots) for Each Group eFigure 5. Mean Slope of Averaged Eyes Visual Field Pattern Standard Deviation (dB/Year; Colored Dots With SE Bars) and Individual Subjects’ Slopes (Gray Dots) for Each Group eFigure 6. Mean Slope of Averaged Eyes Contrast Sensitivity Score at 12 cpd (Score/Year; Colored Dots With SE bars) and Individual Subjects’ Slopes (Gray Dots) for Each Group eFigure 7. Mean Slope of Groton Maze Learning Test (GMLT) Total Errors (Errors/Year; Colored Dots With SE bars) and Individual Subjects’ Slopes (Gray Dots) for Each Group eFigure 8. Scatter Plot Showing Significant Correlation Between RNFL Thickness Change Over Time (Microns/Year) and mTBI Severity Score for the Whole Sample eReferences [file jamanetwopen-e2030824-s001.pdf]

Supplemental Online Content

Gilmore CS, Lim KO, Garvin MK, et al. Association of optical coherence tomography with longitudinal neurodegeneration in veterans with chronic mild traumatic brain injury. *JAMA Netw Open.* 2020;3(12):e2030824. doi:10.1001/jamanetworkopen.2020.30824

eAppendix. Supplemental Methods
eFigure 1. Frequency Distribution of the Time, in Years, Participants Were Enrolled in the Study
eFigure 2. Frequency Distribution of the Number of Time Points of Data Collected for Each Participant
eFigure 3. Frequency Distribution of the Number of Self-reported Blast (3a) and Nonblast (3b) Subconcussive Head Injury Exposures in the TBI Group
eFigure 4. Mean Slope of Averaged Eyes Visual Field Mean Deviation (dB/year; Colored Dots With SE Bars) and Individual Subjects' Slopes (Gray Dots) for Each Group
eFigure 5. Mean Slope of Averaged Eyes Visual Field Pattern Standard Deviation (dB/Year; Colored Dots With SE Bars) and Individual Subjects' Slopes (Gray Dots) for Each Group
eFigure 6. Mean Slope of Averaged Eyes Contrast Sensitivity Score at 12 cpd (Score/Year; Colored Dots With SE bars) and Individual Subjects' Slopes (Gray Dots) for Each Group
eFigure 7. Mean Slope of Groton Maze Learning Test (GMLT) Total Errors (Errors/Year; Colored Dots With SE bars) and Individual Subjects' Slopes (Gray Dots) for Each Group
eFigure 8. Scatter Plot Showing Significant Correlation Between RNFL Thickness Change Over Time (Microns/Year) and mTBI Severity Score for the Whole Sample
eReferences

This supplemental material has been provided by the authors to give readers additional information about their work.

© 2020 Gilmore CS et al. *JAMA Network Open.*

eAppendix. Supplemental Methods

1. Participant Recruitment and Study Timeline

Participants were recruited from two sources:
1) an ongoing pilot study investigating longitudinal neurodegeneration in Veterans with mild TBI. Participants enrolled in that study were screened via chart review to see if they were eligible for the current DoD and VA Chronic Effects of Neurotrauma Consortium (CENC) study (e.g. they had no contraindications for the MRI scans which were added at the time of CENC funding). If they were eligible, they were approached about participating in the study. If they decided to enroll, their data from the pilot study were included in longitudinal analyses. Number of subjects recruited from this pilot study: 45.
2) We obtained a list of patients seen in Primary Care clinics at the Minneapolis VA Health Care System (MVAHCS) between 2015-2016. We excluded those ineligible due to age (see below, age 18-65). We sorted the list by county and sent out recruitment letters. If a participant called due to interest in participating, we would screen them over the phone to see if they reported a history of TBI (or not, for Controls). We would then get their permission to screen their medical records for eligibility. Number of subjects recruited this way: 94.
In total, 352 potential participants were screened, 139 were enrolled.

Specific Inclusion/Exclusion Criteria for the study were:
Inclusion Criteria: 1) Veterans receiving services through the MVAHCS, 2) Diagnosed with symptomatic or mild TBI per Mayo TBI Severity Classification System[9], 3) age 18 to 65 years, 4) Ambulatory and living independently.
Same criteria for Control participants, except they had no history of TBI or head trauma.

Exclusion Criteria: 1) Moderate or severe TBI (due to constraints imposed by DOD/VA funding RFA), 2) Current suicidal or homicidal ideation with intent and/or plan that, in the judgment of the investigator, should be the focus of treatment, 3) Severe cognitive impairment, 4) Unable to comprehend or communicate in English, 5) Diagnosis of multiple sclerosis (due to the impact of this disease on the eye), 6) Long-term use of opioid (or enrollment in the Narcotics Renewal Program), 7) Any chemotherapy or radiation therapy (applied to the head/neck area), 8) Congenital/genetic brain disorders (e.g., Fetal Alcohol Syndrome, FAS) or non-traumatic brain injury (e.g., tumor or stroke), 9) Severe mental health diagnosis (including bipolar, schizoaffective, schizophrenia, and schizotypal or borderline personality disorder), 10) Any eye condition that prevents collection of OCT images or that showed evidence of retinal thinning, thickening or abnormality at the time of entry exam. This would include significant cornea or lens opacities, moderate to severe diabetic retinopathy or maculopathy, significant macular degeneration causing visual acuity worse than 20/25, glaucoma or other optic neuropathy, and 11) Contraindications to MRI (e.g., unapproved metal implants, shrapnel, claustrophobia, pacemaker).

Timeline of study:

- Date of earliest data collection: December, 2012; This was the start of the longitudinal neurodegeneration pilot study.
- Date of first data collection of CENC funded study: June, 2016
- Date of final data point included in analyses: January, 2019
- Data collected every 3-6 months. 6 month visits were "required", 3 month visits were optional.

Figures eFigure 1 and eFigure 2 show frequency distributions of 1) the time the participants were in the study (eFigure 1) and 2) the number of time points of data each participant had (eFigure 2). The mTBI and Control groups did not significantly differ on either of these measures (see those Figures for Wilcoxon rank sum test results).

2. Mild TBI determination, severity scoring, and group characteristics

Ratings of mTBI likelihood and severity were assigned by consensus of doctoral level clinical neuropsychologists based on information secured by trained study interviewers using the semistructured Minnesota Blast Exposure Screening Tool (MN-BEST)[1]. Symptoms of mTBI assessed included altered consciousness (e.g., confusion and disorientation), loss of consciousness (LOC) less than 30 min, post-traumatic amnesia (PTA) up to 24 h, and neurological symptoms (e.g., headache, tinnitus, nausea, sensitivity to light or noise) immediately after the event. The three most significant potential blast-related and impact head injury events were considered, each of which received a severity score ranging from 0 (no concussion) to a potential maximum of 30 (severe TBI). No score was higher than 4 (the maximum within the mTBI range) for a single event in the current sample. The severity scores for each of the reported head injury events were summed to get an overall mTBI severity score for each participant.

As part of the MN-BEST, participants were asked to self-report how many times they have "hit your head hard enough to become unconscious AND/OR experience memory loss" and "hit your head hard enough to feel lasting effects or had a concussion (without any loss of consciousness or post-traumatic amnesia)". Participants' responses to these questions that did not qualify as mild TBIs, were a proxy to the number of repetitive subconcussive head traumas the participants have experienced. Due to time constraints, the source of these subconcussive events is unknown. eFigures 3a and 3b show frequency distributions of the number of self-reported blast (3a) and non-blast (3b) subconcussive head injury exposures in the mTBI group. Looking at the relationship between our main outcome measure, RNFL slope, and each of these measures showed no significant correlation with either the number of blast exposures ($\rho=0.16$, $p=0.22$) or non-blast exposures ($\rho=0.02$, $p=0.89$). With regard to the source of the mTBIs in our sample, only 5 of

© 2020 Gilmore CS et al. *JAMA Network Open.*

the 69 mTBI participants reported experiencing blast-related mTBIs, and all 5 of those participants also reported non-blast mTBI events. Thus, with the current sample, we cannot differentiate effects of blast vs non-blast TBI.

3. Optical Coherence Tomography

OCT imaging was performed in each eye without pupil dilation using the Spectralis OCT1 (Eye Explorer software version 1.9, Heidelberg Engineering, Heidelberg, Germany). Proprietary noise reduction of the device reduces the axial A-scan resolution to a digital resolution of 3.5 μm/pixel. In this, a 360° peripapillary RNFL scan contains 1536 A-scans which provides a transverse digital resolution of about 5 μm in a standard eye. The device obtains up to 40,000 A-scans per second and simultaneously images the fundus with an infrared confocal scanning laser ophthalmoscope (SLO). The real-time eye movement tracking system (ART) uses features in the SLO image, including blood vessels and the optic disc to stabilize the scan on the same location of retina, which minimizes motion artifacts during image acquisition. After definition of a baseline scan as reference, the tracking system enables image acquisition at the same retinal location throughout each follow-up examination. Peripapillary circle scans with a diameter of 12° were obtained in the high-resolution mode. Participants were asked to look at the nasally projected internal fixation light, and the scanning circle was placed concentrically around the optic disc. The Spectralis segmentation software provided the retinal nerve fiber layer (RNFL) thickness at each location along the circular scan. The average RNFL thickness over the 360 degree circular scan that was output to the Spectralis OCT report was used for determining RNFL thickness at each time point for each eye in this prospective study. For macula imaging, the scan protocol captured the central 20° × 15° centered on the fovea with a minimum of 25 B scans per volume. The Iowa Reference Algorithms (Retinal Image Analysis Lab, Iowa Institute for Biomedical Imaging, Iowa City, IA) were used to segment the macula volume OCT scans to derive the thickness of the ganglion cell-inner plexiform layer (GCL-IPL) complex.

Participants were also tested, when possible, by OCT imaging using SD-OCT (Cirrus 4000, Cirrus software version 6.0; Carl Zeiss Meditec, Dublin, CA, USA). Images with signal strength less than 7 were excluded. The peripapillary RNFL thickness measurements were obtained using the optic disc cube 200 × 200 pixel volume scan protocol that covered the 6 × 6 mm^2 area centered on the optic disc. In most cases, the Spectralis OCT average RNFL thickness was used for each time point. However, in a minority of cases, the Spectralis OCT was not available for testing, so on those dates the Cirrus OCT average RNFL value was used after segmenting the disc volume scan in three dimensions using the Iowa Reference Algorithms. We determined that there was an extremely high correlation between the Spectralis and Cirrus OCT average RNFL measurement in subjects that were tested by both instruments on the same day (r = .94). The macula volume scans were obtained using the macular cube 200 × 200 pixel protocol centered on the fovea.

Commercially available segmentation algorithms to assess the GCL-IPL complex use two dimensional analysis of B scans and are sometimes prone to failure. In addition, each OCT manufacturer has its own proprietary algorithm making it difficult to compare results across instruments. This problem can be overcome by using the Iowa Reference Algorithm, a fully three-dimensional (3D), automated algorithm[2–5], which can accurately measure the macular GCL-IPL complex and the RNFL thickness. The incorporation of 3D information allows the Iowa Reference Algorithm to decrease segmentation errors[2–4]. The boundaries of the macular GCL-IPL were defined by the junction between the retinal nerve fiber and ganglion cell layers and the junction between the inner plexiform and inner nuclear layers. The automated segmented layers were inspected for errors and manually corrected if present.

4. Contrast Sensitivity

Threshold for contrast sensitivity was determined for each eye using the Functional Vision Analyzer (FVA) system which utilizes the Functional Acuity Contrast Test (F.A.C.T.; Stereo Optical, Chicago, IL). The device presented sinusoidal gratings in the form of Gabor patches to the respondent. For each spatial frequency, a series of nine patches were presented with a 0.15 log unit or 50% loss of contrast between consecutive patches. Gratings were tilted to the left (+15°), right (−15°) or upright (0°) to keep them within the bandwidth of the visual channel. The respondent indicated the direction of grating tilt for each patch without guessing. The contrast sensitivity score corresponds to the contrast of the last grating that was accurately identified on each row. If a participant could identify no grating on a particular row, the FACT numerical score was zero for that row. Thus, for each participant, we collected 5 contrast sensitivity measures, one for each spatial frequency.

5. Visual Field

The visual field is the portion of space which is visible when gaze is fixed in one direction. The visual field was tested with the Humphrey Visual Field Analyzer (Zeiss Meditec, Dublin, CA), using a 10-2 threshold VF testing pattern. The 10-2 pattern assesses 68 points, all two degrees apart, just one degree from either side of the horizontal and vertical meridians. During this test, the participant is required to press a button when they detect the presence of a small (0.431 diameter), brief (200 ms) test light presented on a background (10 candelas/m2). The participant is asked to maintain the same eye position by fixating on a small centrally located fixation light. Different locations within a given region of the visual field are tested until the threshold, or the stimulus intensity seen 50% of the time, is seen at each test location. The outcome measures are 1) mean deviation and 2) pattern standard deviation reported in decibels (dB). Mean deviation (MD) is the mean deviation in the participant's results compared to those expected from the age-matched normative database, with values becoming more negative as the overall field worsens. Pattern standard deviation (PSD) is a depiction of focal defects. It is determined by comparing the differences between adjacent points. Higher values represent more focal losses, while lower values can represent either no loss or diffuse loss.

© 2020 Gilmore CS et al. *JAMA Network Open.*

The central 10 degrees of visual field was tested in anticipation of being able to correlate any change over time with changes in the central 8 degrees of retinal GCL-IPL thickness, the reduced variability compared to threshold testing in locations peripheral to 10 degrees, the denser sampling within the central 10 degrees afforded by the 10-2 program, and the ease of testing for the subjects within their central 10 degrees with FDP.

6. Cogstate Tasks

Cognitive ability was measured using five tests from the Cogstate battery (Cogstate Ltd., New Haven, CT)[6]: 1) Detection, a test of reaction time that assesses psychomotor processing speed. Participants quickly right-click on the mouse when a playing card turns face up. 2) Identification, a reaction time test that assesses attention. Participants identify whether the card presented is red. 3) One-Back Task assesses working memory. Participants identify whether the card currently face-up is identical to the previous card. 4) One Card Learning assesses short-term visual learning and memory. Participants identify whether the card face-up has ever appeared before during the task. Six repeating playing cards are interspersed with eight non-repeating cards, for a total of 14 different cards across the 42 trials of this task. 5) Groton Maze Learning Test (GMLT) measures executive function using a maze learning paradigm. A 10 x 10 grid of tiles is presented on the screen. A 28-step pathway is hidden among these tiles. The participant must move one step at a time from the start toward the end by touching a tile next to their current location. If the correct move is made a green checkmark appears and if the move is incorrect a red cross is revealed. Total number of errors made during the GMLT is the outcome measure. Reaction time is the outcome measure for the other Cogstate tasks, quantified as the mean of the log10 transformed reaction times for correct responses.

7. Cook's Distance outlier determination

The Cook's Distance method of identifying outliers was used[7]. Cook's Distance (D) measures how much influence each single data point has on the overall fitted model. Within each individual participant's retinal thickness data, for each single observation (i.e. single time point of data), Cook's Distance measured the change in the fitted model across all observations with and without including that observation, so that we know how much that observation impacted the fitted values. If a single observation's D value was greater than four times the mean D across all observations, that data point was considered an outlier, removed so as to not have undue influence on the model, and the linear regression model fit to the remaining RNFL or GCL-IPL data points.

8. False Discovery Rate

We chose to control the false discovery rate (FDR), the proportion of "discoveries" (significant results) that are actually false positives, using the technique developed by Benjamini and Hochberg[8]. The false discovery rate was set at 0.25, which indicates that we are willing to accept up to 25% of the tests with significant results being false positives. For the False Discovery Rate (FDR) procedure, the individual p-values are put in order from smallest to largest. The smallest p-value has a rank of $i=1$, the next smallest has rank $i=2$, etc. Each individual p-value is compared to its Benjamini-Hochberg critical value, $(i/m)Q$, where i is the rank, m is the total number of tests, and Q is the false discovery rate. The largest p-value with $p < (i/m)Q$ is significant, and all of the p-values smaller than it are also significant, even the ones that aren't less than their critical value. With five tests at an FDR of 0.25, the critical values are 0.05, 0.10, 0.15, 0.20, and 0.25.

© 2020 Gilmore CS et al. *JAMA Network Open.*

eFigure 1. Frequency Distribution of the Time, in Years, Participants Were Enrolled in the Study.

Median time for the mTBI group was 1.8 years and 1.6 years for the Controls. There was no significant difference between groups (Wilcoxon rank sum test: W=2798, p=0.08).

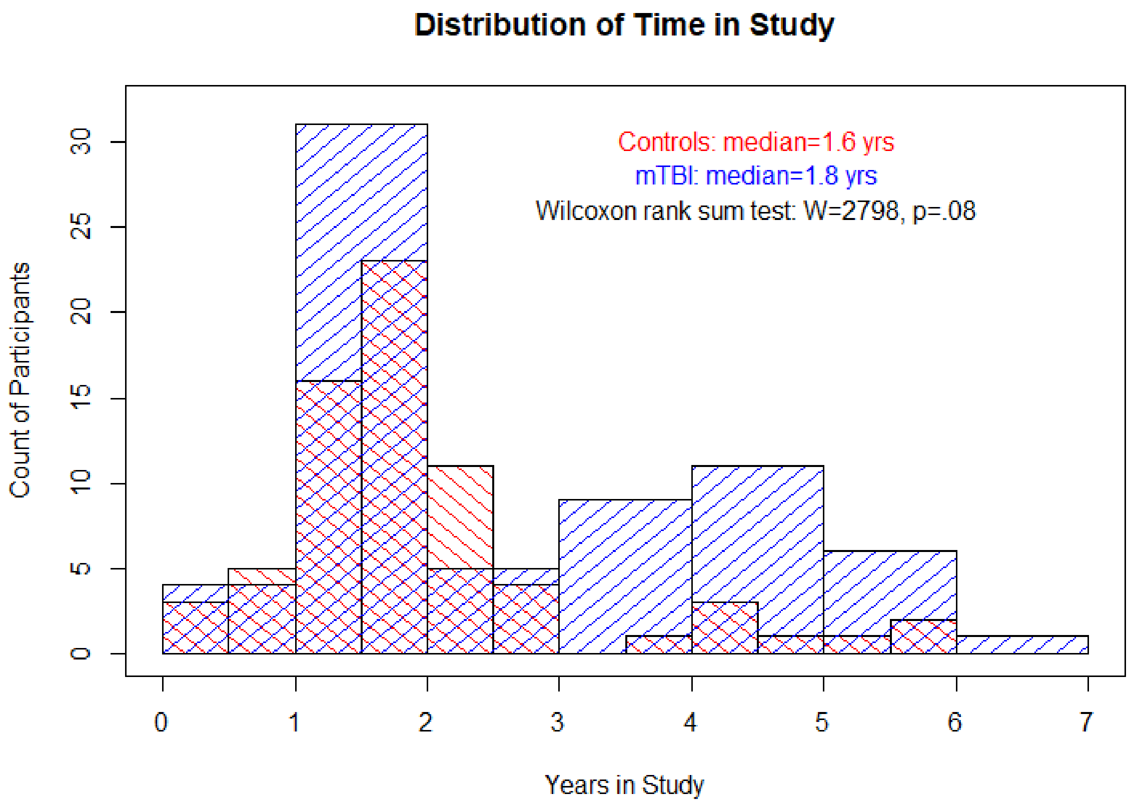

© 2020 Gilmore CS et al. *JAMA Network Open.*

eFigure 2. Frequency Distribution of the Number of Time Points of Data Collected for Each Participant.
Median number of data points for the mTBI group was 4 and for the Controls was 5. There was no significant difference between groups (Wilcoxon rank sum test: W=2260, p=0.71).

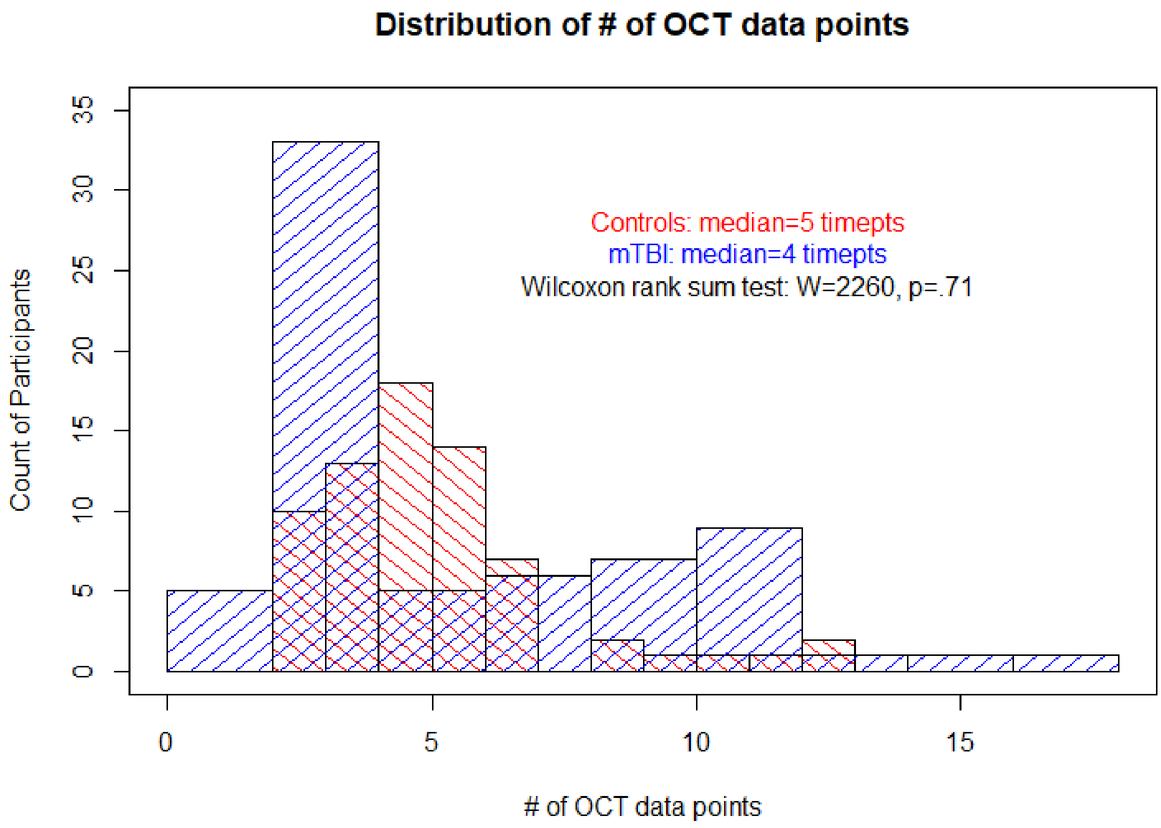

© 2020 Gilmore CS et al. *JAMA Network Open.*

<u>eFigure 3</u>. Frequency Distribution of the Number of Self-reported Blast (3a) and Nonblast (3b) Subconcussive Head Injury Exposures in the TBI Group.

3a.

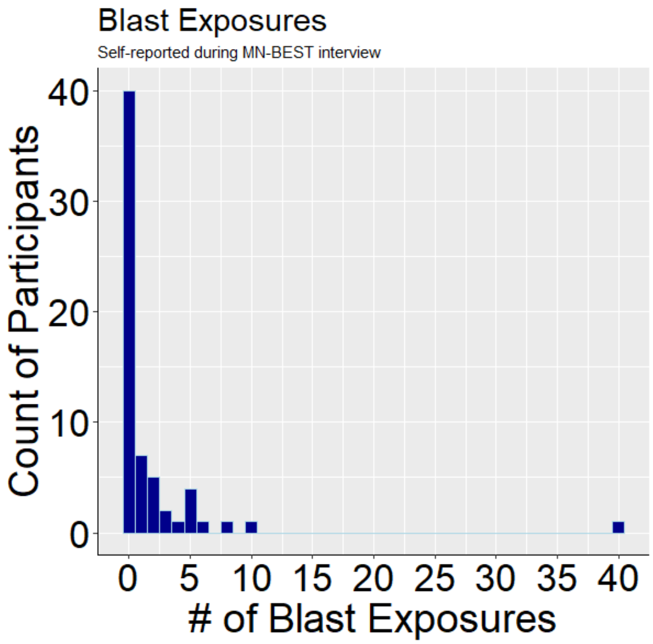

3b.

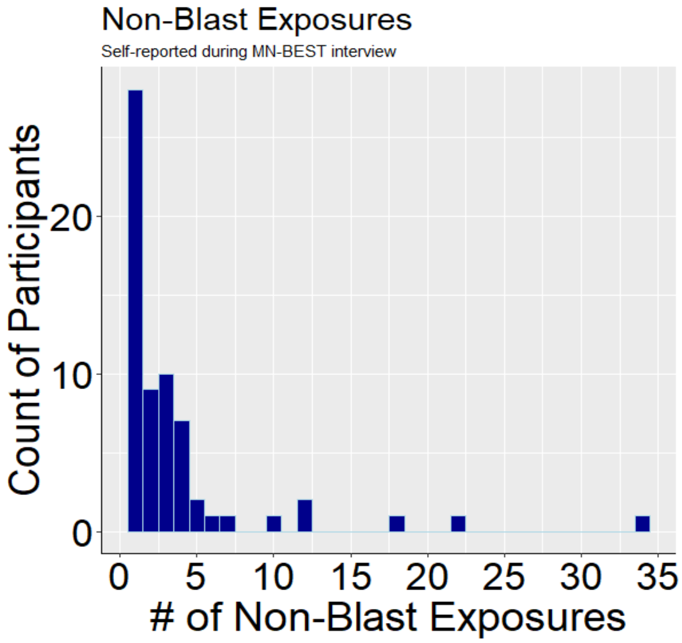

© 2020 Gilmore CS et al. *JAMA Network Open.*

<u>eFigure 4</u>. Mean Slope of Averaged Eyes Visual Field Mean Deviation (dB/year; Colored Dots With SE Bars) and Individual Subjects' Slopes (Gray Dots) for Each Group. Groups significantly differ: p = 0.046, Cohen's d = 0.36.

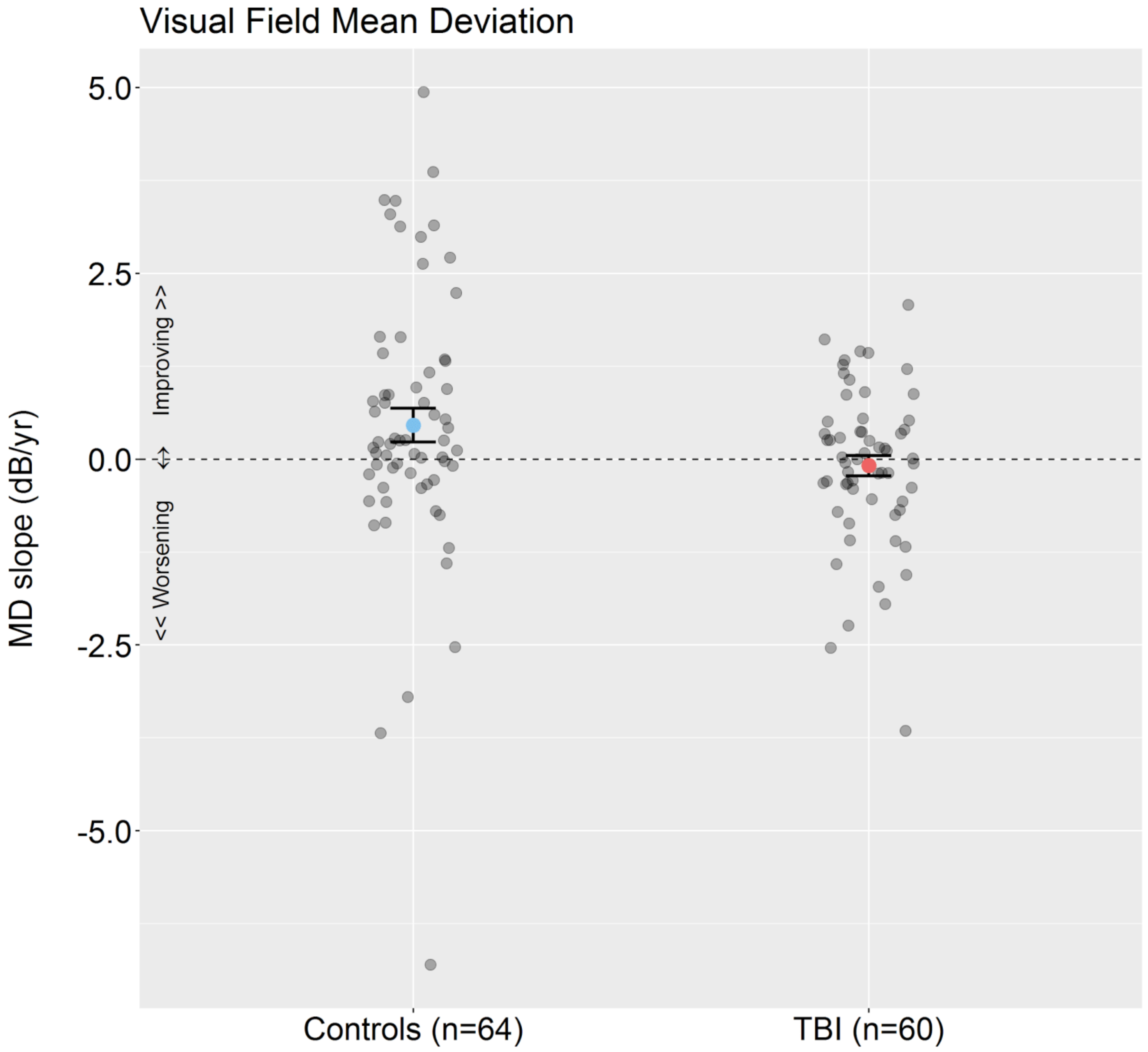

© 2020 Gilmore CS et al. *JAMA Network Open.*

<u>eFigure 5</u>. Mean Slope of Averaged Eyes Visual Field Pattern Standard Deviation (dB/Year; Colored Dots With SE Bars) and Individual Subjects' Slopes (Gray Dots) for Each Group. Groups significantly differ: p = 0.03, Cohen's d = 0.39.

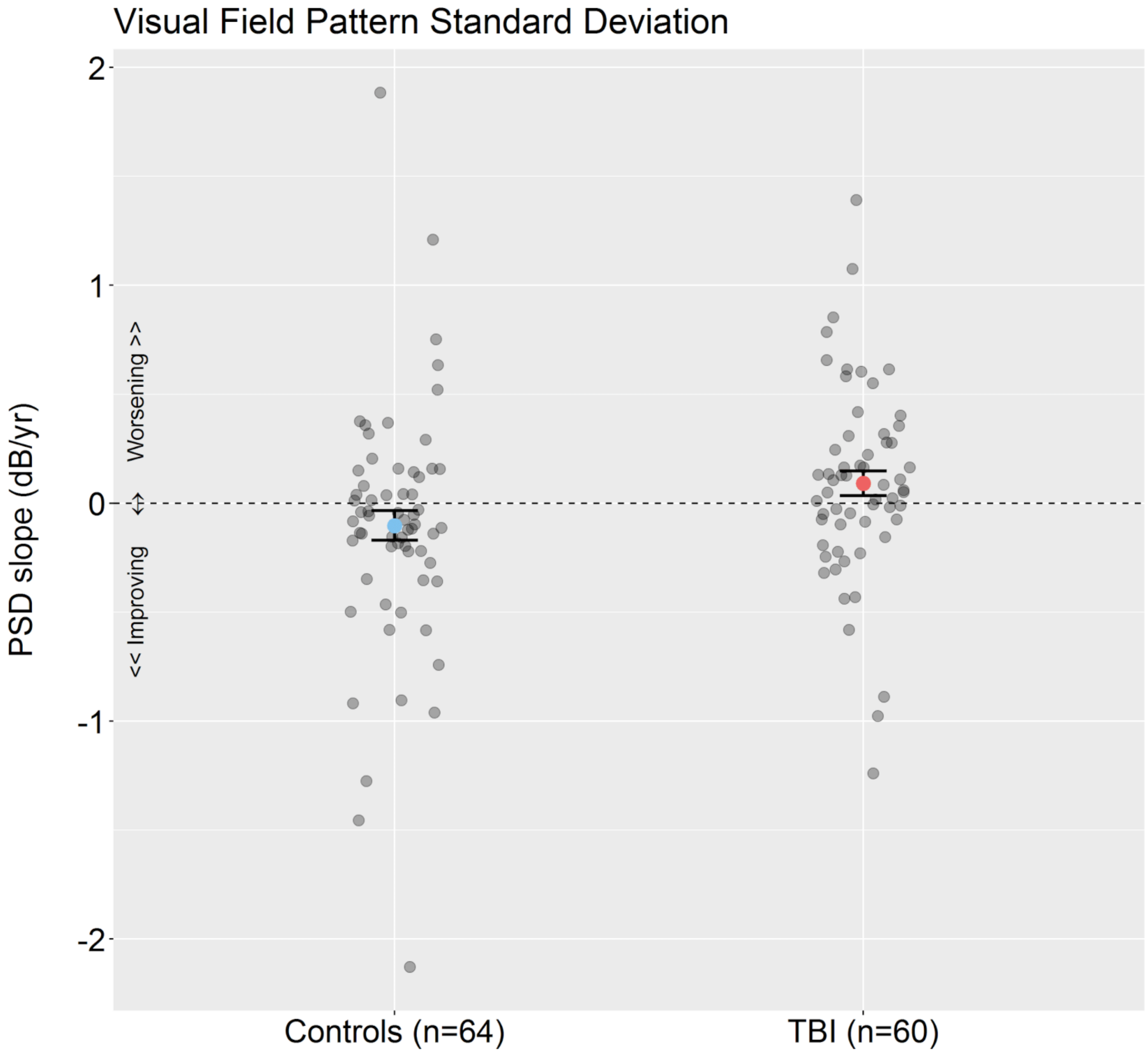

© 2020 Gilmore CS et al. *JAMA Network Open.*

<u>eFigure 6</u>. Mean Slope of Averaged Eyes Contrast Sensitivity Score at 12 cpd (Score/Year; Colored Dots With SE bars) and Individual Subjects' Slopes (Gray Dots) for Each Group. Groups significantly differ: p = 0.02, Cohen's d = 0.43.

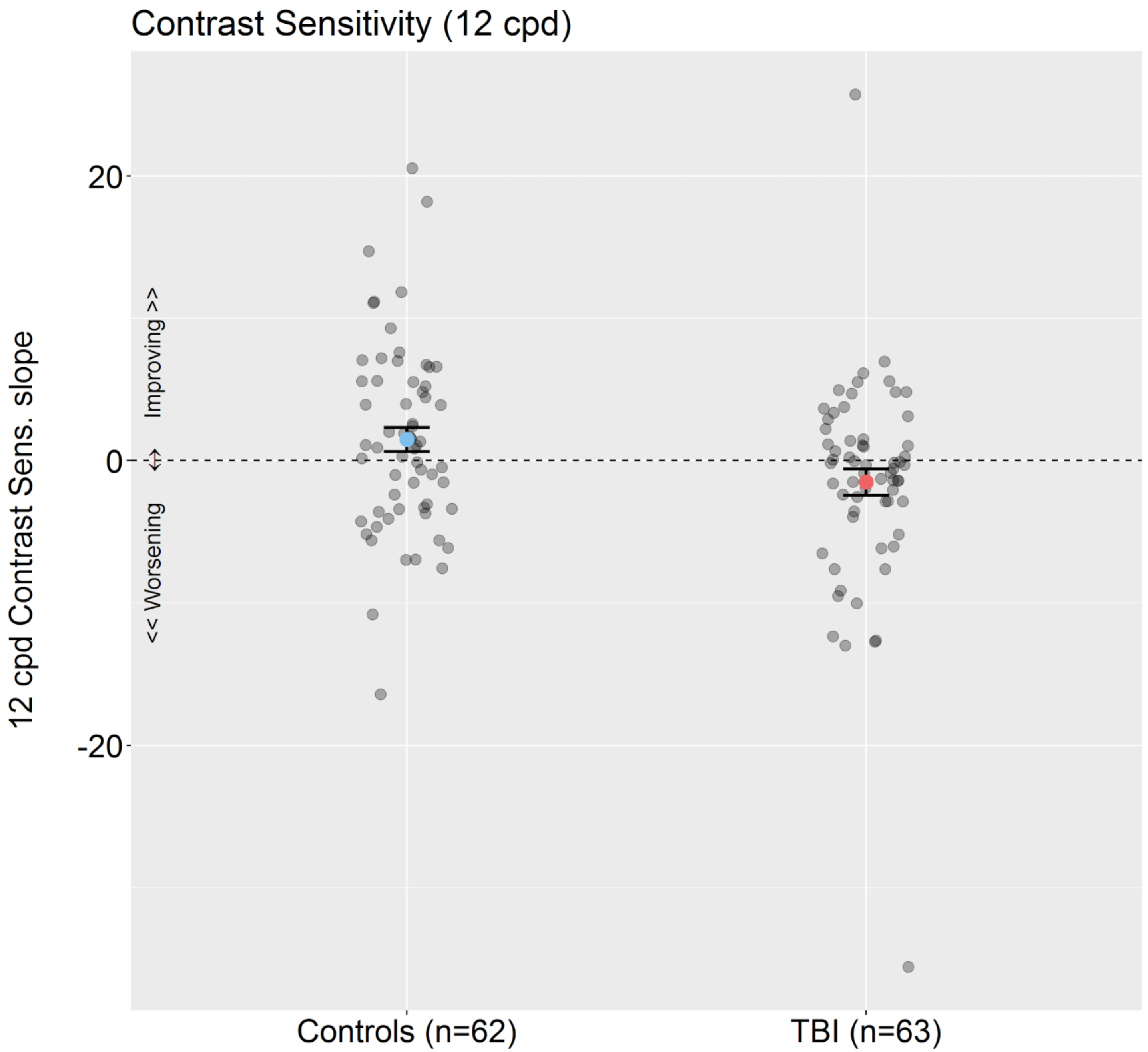

© 2020 Gilmore CS et al. *JAMA Network Open.*

eFigure 7. Mean Slope of Groton Maze Learning Test (GMLT) Total Errors (Errors/Year; Colored Dots With SE bars) and Individual Subjects' Slopes (Gray Dots) for Each Group. Groups significantly differ: p = 0.04, Cohen's d = 0.37.

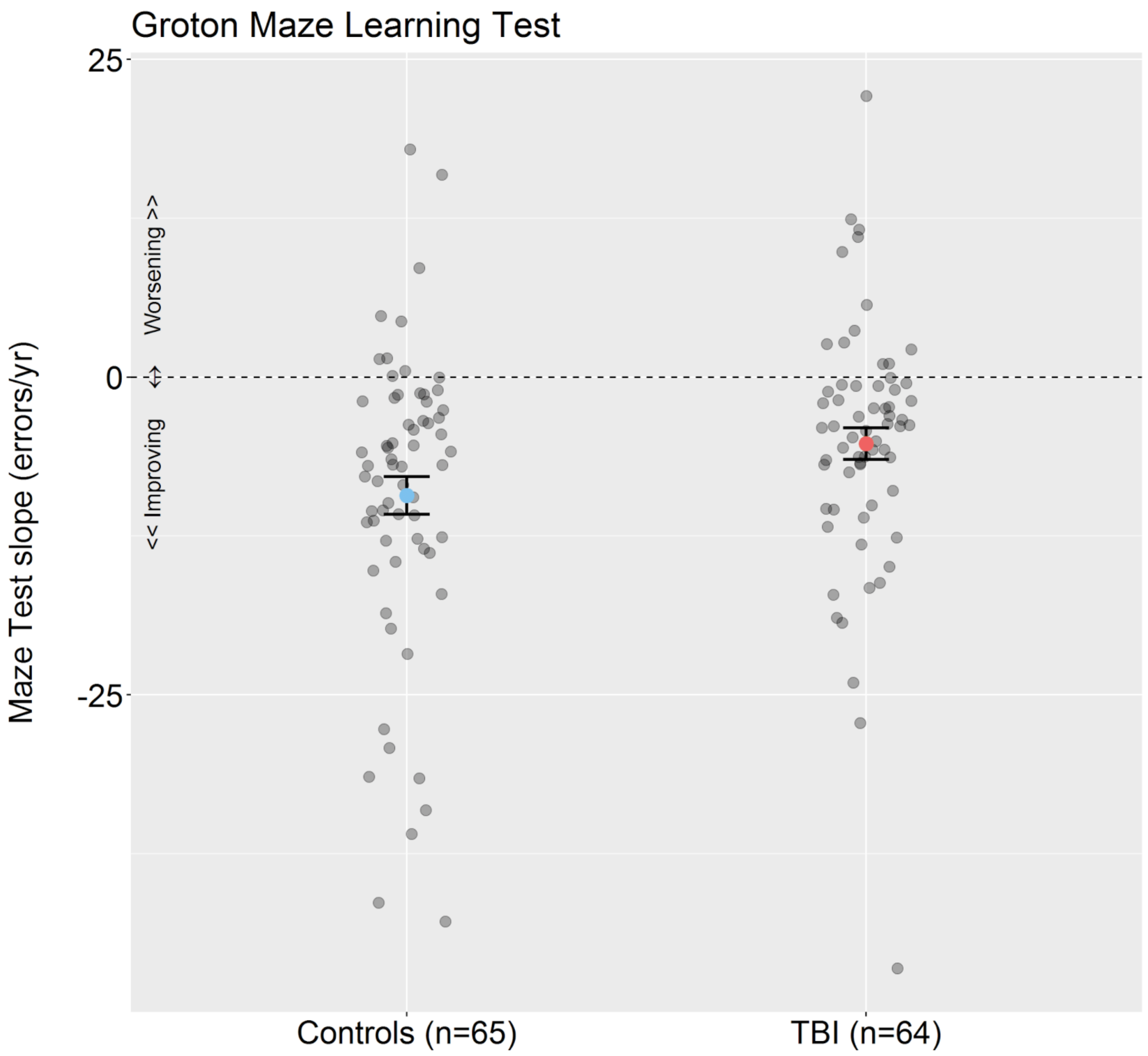

© 2020 Gilmore CS et al. *JAMA Network Open.*

<u>eFigure 8</u>. Scatter Plot Showing Significant Correlation Between RNFL Thickness Change Over Time (Microns/Year) and mTBI Severity Score for the Whole Sample.

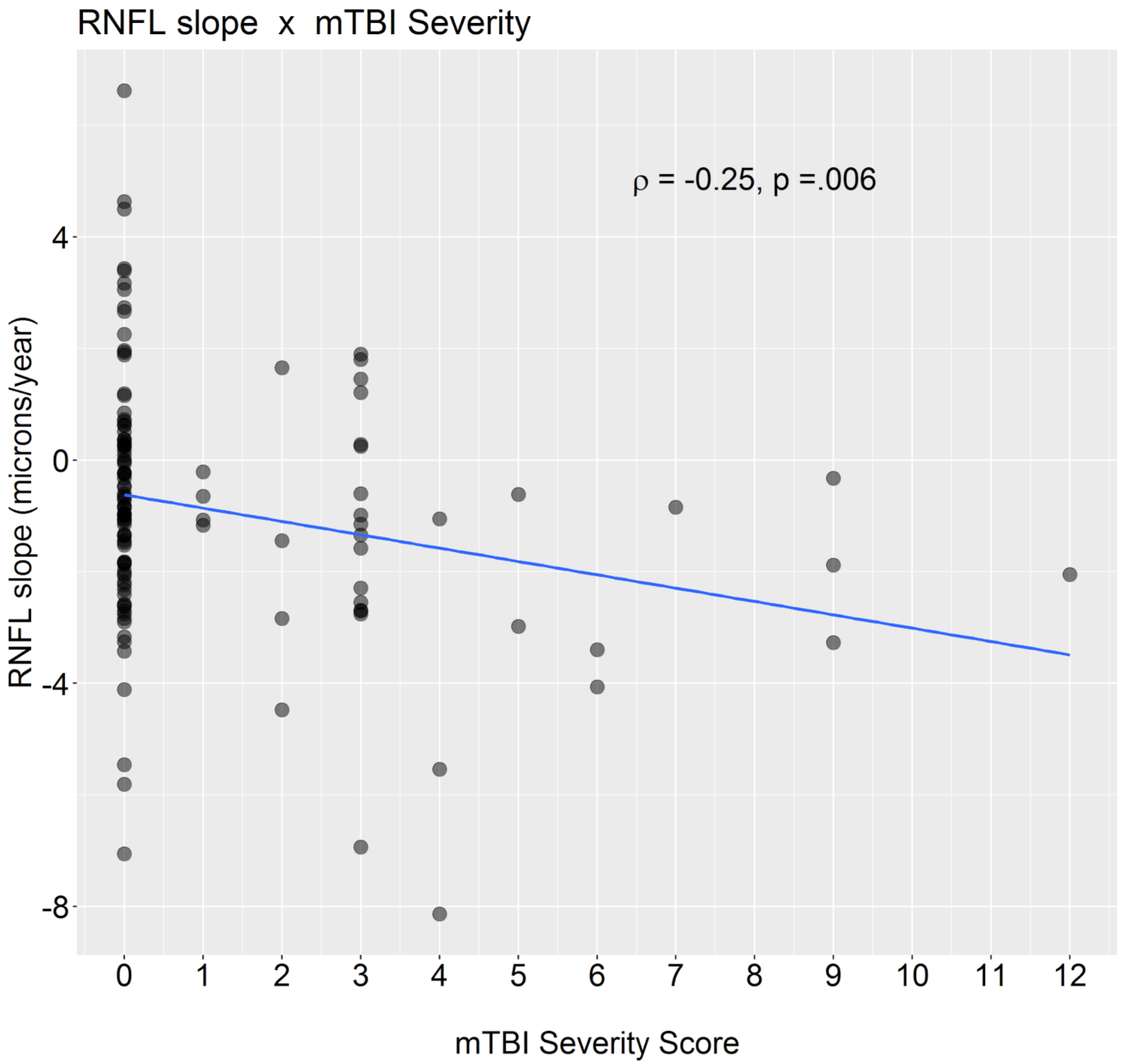

© 2020 Gilmore CS et al. *JAMA Network Open.*

eReferences

1. Nelson NW, Hoelzle JB, McGuire KA, Ferrier-Auerbach AG, Charlesworth MJ, Sponheim SR. Neuropsychological evaluation of blast-related concussion: illustrating the challenges and complexities through OEF/OIF case studies. *Brain Inj*. 2011;25(5):511-525.

2. Lee K, Niemeijer M, Garvin MK, Kwon YH, Sonka M, Abramoff MD. Segmentation of the optic disc in 3-D OCT scans of the optic nerve head. *IEEE Trans Med Imaging*. 2010;29(1):159-168.

3. Garvin MK, Abramoff MD, Kardon R, Russell SR, Wu X, Sonka M. Intraretinal layer segmentation of macular optical coherence tomography images using optimal 3-D graph search. *IEEE Trans Med Imaging*. 2008;27(10):1495-1505.

4. Garvin MK, Abràmoff MD, Wu X, Russell SR, Burns TL, Sonka M. Automated 3-D intraretinal layer segmentation of macular spectral-domain optical coherence tomography images. *IEEE Trans Med Imaging*. 2009;28(9):1436-1447.

5. Wang J-K, Kardon RH, Kupersmith MJ, Garvin MK. Automated quantification of volumetric optic disc swelling in papilledema using spectral-domain optical coherence tomography. *Invest Ophthalmol Vis Sci*. 2012;53(7):4069-4075.

6. Maruff P, Thomas E, Cysique L, et al. Validity of the CogState brief battery: relationship to standardized tests and sensitivity to cognitive impairment in mild traumatic brain injury, schizophrenia, and AIDS dementia complex. *Arch Clin Neuropsychol*. 2009;24(2):165-178.

7. Cook RD. Influential Observations in Linear Regression. *J Am Stat Assoc*. 1979;74(365):169-174.

8. Benjamini Y, Hochberg Y. Controlling the False Discovery Rate: A Practical and Powerful Approach to Multiple Testing. *Journal of the Royal Statistical Society Series B (Methodological)*. 1995;57(1):289-300.

9. Malec JF, Brown AW, Leibson CL, et al. The mayo classification system for traumatic brain injury severity. J Neurotrauma. 2007;24(9):1417-1424.

© 2020 Gilmore CS et al. *JAMA Network Open.*
